# Criteria of “persistent vomiting” in the WHO 2009 warning signs for dengue case classification

**DOI:** 10.1186/s41182-016-0014-9

**Published:** 2016-05-16

**Authors:** Nguyen Lam Vuong, Dao Huy Manh, Nguyen Thi Mai, Le Hong Phuc, Van Thuy Luong, Vo Duy Quan, Nguyen Van Thuong, Nguyen Thi Phuong Lan, Cao Thi My Nhon, Shusaku Mizukami, Nguyen Ngoc Doan, Vu Thi Que Huong, Nguyen Tien Huy, Kenji Hirayama

**Affiliations:** University of Medicine and Pharmacy, 217 Hong Bang, District 5, Ho Chi Minh City, 70000 Vietnam; Department of Immunogenetics, Institute of Tropical Medicine (NEKKEN), Leading Graduate School Program, and Graduate School of Biomedical Sciences, Nagasaki University, Nagasaki, Japan; Department of Immunology and Microbiology, Pasteur Institute Ho Chi Minh City, Ho Chi Minh City, Vietnam; Nguyen Dinh Chieu Hospital, Ben Tre, Ben Tre Province Vietnam; Children’s Hospital No.1, Ho Chi Minh City, Vietnam; Department of Clinical Product Development, Institute of Tropical Medicine (NEKKEN), Leading Graduate School Program, and Graduate School of Biomedical Sciences, Nagasaki University, Nagasaki, Japan

**Keywords:** Dengue, Vomiting, Warning sign, Severity, Prediction

## Abstract

**Introduction:**

Dengue is a viral disease that spreads rapidly in the tropic and subtropic regions of the world and causes 22,000 deaths annually. In 2009, the World Health Organization (WHO) released a new classification of dengue infections, which divided them into three categories: dengue without warning sign (D), dengue with warning sign (DWS), and severe dengue (SD). However, researchers have been using different criteria to define persistent vomiting; therefore, we aimed to evaluate the ability of the number of vomiting times in early prediction of SD development among D/DWS patients.

**Method:**

A hospital-based cohort study was conducted in Ben Tre-south of Vietnam. We enrolled confirmed dengue patients with D and DWS at admission. The final classification was determined on the discharged day for every patient based on the classification of WHO 2009 without using vomiting symptom, using the receiver operating characteristic (ROC) curve to evaluate the ability of the number of vomiting times in early prediction of SD development among D/DWS patients.

**Result:**

The prevalence of vomiting symptom was higher in SD group than D/DWS group (92 versus 46 %, *p* = 0.006), and the median of the number of vomiting times was higher in SD group than D/DWS group (2.5 versus 0, *p* = 0.001). To distinguish SD from D/DWS, the ROC curve of the number of vomiting episodes showed that the area under the curve was 0.77; with the cut point of two, the sensitivity and specificity were 92 and 52 %, respectively.

**Discussion:**

The number of vomiting times could be a good clinical sign which can early predict SD from the group of D/DWS. We suggest the definition of persistent vomiting should be vomiting two times or more per day.

## Introduction

Dengue is a re-emerging vector-borne viral disease that spreads rapidly in the tropic and subtropic regions of the world, caused by dengue virus. The World Health Organization (WHO) estimates a mortality rate of dengue infection between 1 and 5 %, mainly among children with shock. Among 500,000 cases of dengue hemorrhagic fever, 22,000 deaths occur annually [1–3]. It is estimated that more than 390 million dengue virus infections occur annually worldwide, but only one fourth are clinically apparent [4].

The classification of 2009 WHO guideline of dengue infections into three categories (dengue without warning sign (D), dengue with warning sign (DWS), and severe dengue (SD)) has clear advantages in research, epidemiology, and clinical use [5]. Early detection of the individuals who will develop SD among D/DWS patients is very important for timely clinical management. However, among the warning signs, the literature and the 2009 WHO guideline of dengue infections do not provide a clear criterion of persistent vomiting. Many authors used different criteria to define persistent vomiting, like more than five times in 6 h or more than three times in 1 h, or vomiting during two or more consecutive days, or more than three episodes of vomiting in 12 h [6–8]. Hence, we conducted a study in Vietnam to evaluate the ability of the number of vomiting episodes in early prediction of SD development among D/DWS patients and then to suggest a criterion for persistent vomiting.

## Methods

This was a hospital-based cohort study taking place in Nguyen Dinh Chieu hospital in Ben Tre province, south of Vietnam, from June 2011 to November 2013. The study was approved by Institutional Review Board of Institute of Tropical Medicine (NEKKEN), Nagasaki University (No.11063072), and the Pasteur Institute in Ho Chi Minh City (PIHCM) (No.602/QD-Pas 27/12/10). Informed consent was taken from patients, and if the patient was under 18 years old, informed consent was given by a parent or guardian. Patients were eligible for enrollment when they were hospitalized with diagnosis of dengue fever, acute onset fever (≥38 °C) for less than 72 h, and without severe symptoms before hospitalization. Dengue infection was confirmed by positive serologic assays, virus isolation, or reverse transcription polymerase chain reaction (RT-PCR) for the identification of viral serotype, as described in our previous studies [9, 10]. All the signs including the warning signs which are descriptive in the WHO 2009 classification [11] were collected before admission till discharge from the hospital. All the patients were in D/DWS group when admission, and they were followed until discharge to determine whether they would develop to SD. The final diagnosis was determined on the discharged day for every patient based on the classification of WHO 2009 without using vomiting symptom, using the receiver operating characteristic (ROC) curve for these two groups to evaluate the ability of the number of vomiting times in early prediction of SD development among D/DWS patients. Stata 12.0 was used for analyzing the study data.

## Results

**Fig. 1:**
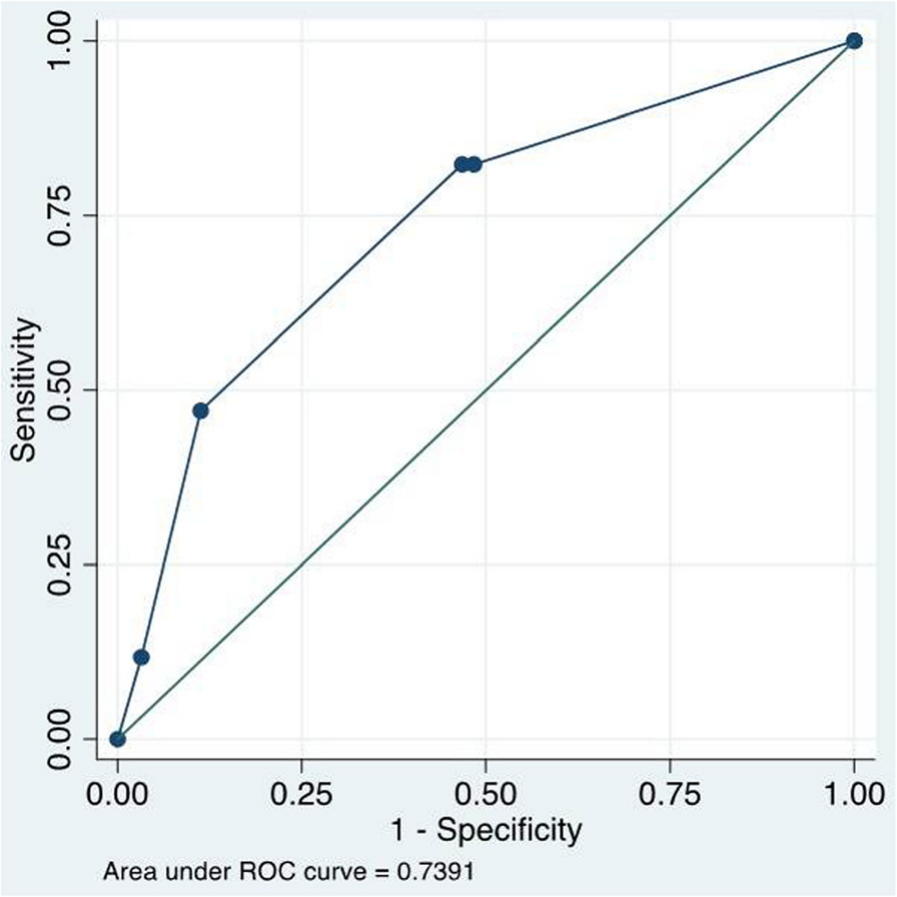
ROC curve of the number of vomiting episodes for predicting SD development among D/DWS patients

There were 79 patients participated in this study. Among them, female ratio was 51 % (40/79); the median (inter quartile range, IQR) of age was 14 (11–19) years. According to the 2009 WHO classification, the patients with final D/DWS/SD diagnosis were 32/35/12 (41, 44, and 15 %, respectively). The most three frequent clinical signs were vomiting (*n* = 44, 56 %), abdominal pain or tenderness (*n* = 35, 44 %), and skin bleeding (*n* = 30, 38 %). Most of the patients had secondary infection (*n* = 63, 80 %). When comparing the vomiting sign in the two groups (D/DWS versus SD), the frequency of vomiting sign was significantly higher among patients in SD group (92 %, 11/12) than among patients in D/DWS group (49 %, 33/67); *p* = 0.013 (chi-square test). In addition, the median (IQR) of the number of vomiting episodes per day was significantly higher in SD group (2.5, 2–3) than in D/DWS group (0, 0–2); *p* = 0.001 (Mann-Whitney test). Figure 1 shows the ROC curve of the number of vomiting episodes per day when predicting SD, the area under the ROC curve is 0.77. In the cut point of two, the sensitivity and specificity were 92 and 52 %, respectively. With this cut point, we had 43 patients (54 %) with persistent vomiting in this study and we found this warning sign to be associated with the development of SD (odds ratio (OR) 12.0; 95 % confident interval (95 % CI) 1.5–532.2).

## Discussion

In this study, vomiting was the most frequent clinical symptom. This is a popular symptom which can be easy to recognize and monitor, even by the patients. When comparing the vomiting sign in the two groups (D/DWS versus SD), SD group had significantly higher prevalence of vomiting sign and also higher median of the number of vomiting episodes. Therefore, vomiting sign could be a good clinical sign which can early predict SD development among D/DWS patients. Indeed, the ROC curve of the number of vomiting episodes per day when predicting SD among D/DWS patients had the good area under the curve, which was 0.77. With good sensitivity, the chosen cut point of the number of vomiting episodes per day is two, of which the sensitivity and specificity are 92 and 52 %, respectively. With this cut-point value, we found the strong association of persistent vomiting with the development of SD (OR 12.0 (95 % CI 1.5–532.2)); this result is similar to Thanachartwet et al. (OR 4.817 (95 % CI 1.375–16.873)) [12]. With patients diagnosed of dengue infection with acute onset fever (≥38 °C) for less than 72 h, when the patient vomits two times or more per day, clinician should carefully follow up him/her because this patient may early turn into SD according to 2009 WHO classification. Therefore, our research suggests a criterion for persistent vomiting should be vomiting two times or more per day. Further, large studies are required to validate this criterion.
